# Scientific writing: how to get published

**DOI:** 10.46989/001c.120878

**Published:** 2024-07-12

**Authors:** Junia Melo, Mohamad Mohty

**Affiliations:** 1 Faculty of Health and Medical Sciences, University of Adelaide, Adelaide, SA 5000, AustraliaUniversity of Adelaide; 2 Sorbonne Université; 3 Centre de Recherche Saint-Antoine INSERM UMRs938; 4 Service d’Hématologie Clinique et de Thérapie Cellulaire, Hôpital Saint Antoine, AP-HP, Paris, France

**Keywords:** publication, impact factor, scientific writing

## Introduction

**Figure 1.**
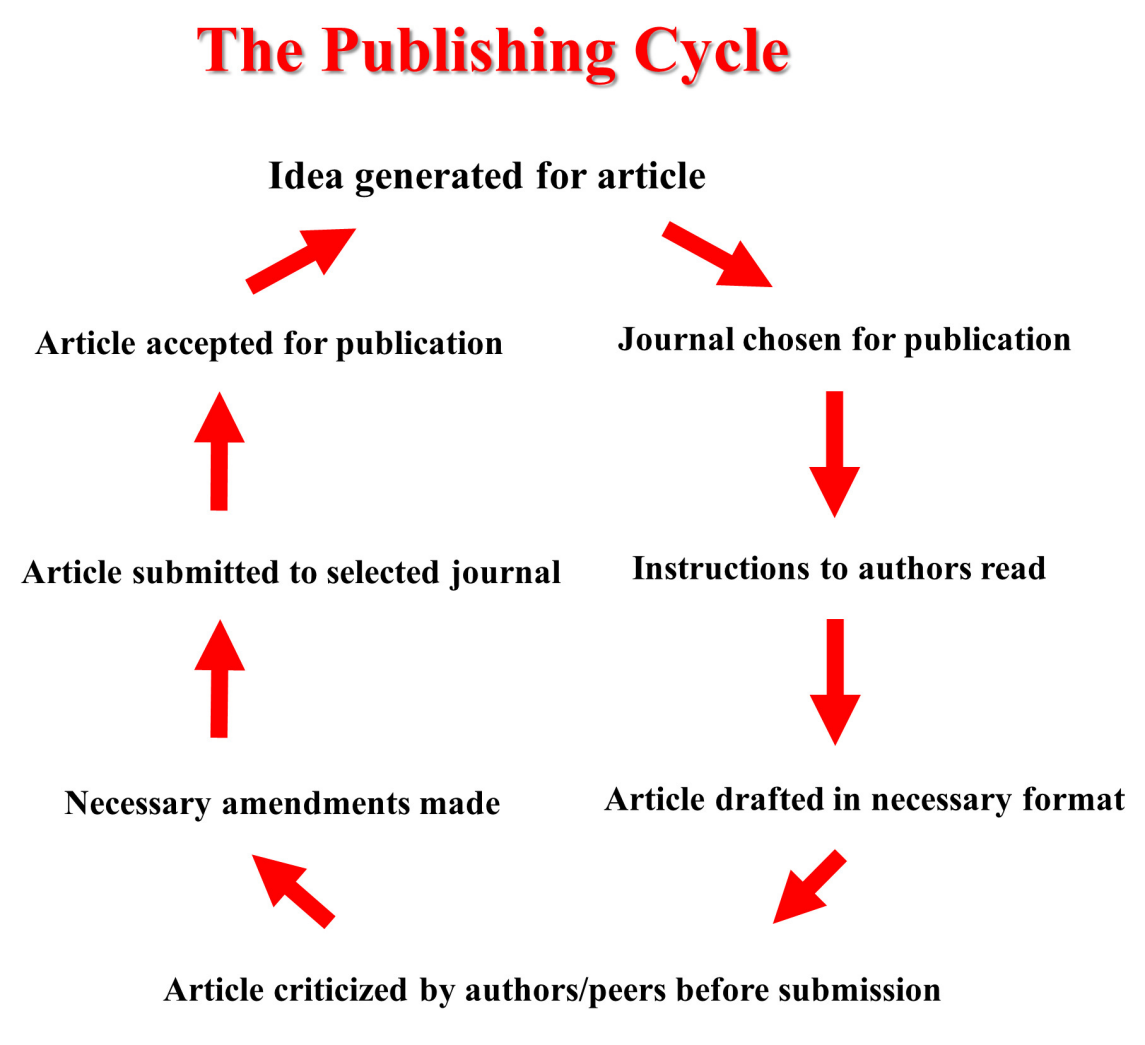
The publishing cycle.

Publishing scientific and medical papers is a key component of an academic career. In addition to furthering one’s own professional development, it is mandatory to share and disseminate knowledge for the benefit of the global community and to advance research. The publishing cycle starts with an idea or some findings one (or several investigators) would like to share with the broader scientific community. Before starting to write a manuscript, a crucial question relates to the novelty of the work. *Novelty* is a recurrent criterion in the publishing cycle (**Figure 1**), as it reflects the importance and potential for publication of the work in a high-level peer-reviewed journal. However, less novel findings can still be published, if they are confirmatory or relate to some challenging research or field, and can contribute to strengthen the available body of literature in a given field.

The type of manuscript to write depends on the amount of work and its significance. A full or original article normally requires substantial significant original research. If it is not, the manuscript should be restricted in length for early and quick disclosure of the findings, and submitted as a ‘Letter to the Editor’ or short/rapid communication, in which it may have a better chance of being accepted. In the case of a literature review, a journal can invite one or several authors to submit such a review or perspective article. Alternatively, one may be able to negotiate such an article with a journal editor in case of a timely and good idea.

The choice of the target journal is another critical issue, because this dictates whether the work can reach an appropriate audience within a reasonable time frame. Making a pre-submission enquiry, before completing the full submission process, may help to better choose the most appropriate journal. An article which does not fulfil the aims and scope of the journal is usually rejected. Also, a report which is too parochial, will not appeal to a wider, international audience, and is likely to be rejected. An article must always provide novelty, or at least investigate a question from a different and new angle. A flawed study design, a suboptimal theoretical framework with lack of relevant references, or a badly prepared manuscript, written in poor English, are all legitimate reasons for rejection.

To be accepted for publication, an article has to provide insight into an important issue. Such insight should be useful in its own right, but can also be used to develop a framework or theory, or stimulate new, important questions. Likewise, the methods used to explore the issue are appropriate, are applied rigorously and have the power to explain why and how the data support the conclusions. Furthermore, well referenced connections to prior work in the field or from other fields are made when discussing the findings, in parallel with highlighting the novel aspects of the work. Embodying all these qualities, a good paper is appealing to read.

## Preparing an article for submission

One cannot overstate the importance of reading and carefully following the “Instructions to Authors” before starting to write a manuscript! Different journals require different formats. Word and/or figure limits need to be respected, and references should be checked for the correct format. Supplemental data may prove to be beneficial and should be included whenever needed and authorized. A cover letter should usually accompany the submission and should be written carefully, because it is often the first (and usually only) piece the Chief Editor reads. Such cover letter should not duplicate the abstract but, rather, state the major theme of the research, and emphasize its *importance* and *novelty.* It may include suggestions for reviewers and/or those with whom a conflict of interest may arise, as well as a statement of financial or other relationships that might lead to a conflict of interest.

A crucial, though often neglected requirement for a good manuscript is its language legibility. No matter how potentially important the data are, if the paper is not written in a concise, accurate manner, it will not be accepted by a good journal. If none of the authors have enough experience and knowledge of the reporting language (almost invariably English, for international readership) and of the academic writing conventions, it is essential to seek professional editing of the manuscript before submission.

## Formatting an article

When formatting an article, the following pitfalls should be avoided, and specific rules should be applied.

**Abstract.** The pitfall is that this is usually too long. An abstract should not overstate findings but rather tell a concise story which is easy to read and take in. When possible, it should provide specifics, but avoiding clogging details (e.g. complex statistical information inside interminable brackets!).

**Introduction.** The pitfall is that it is usually too long. This part should set the stage, state the objectives, provide a brief rationale for the question being investigated, and end with a “tantalizing” phrase, *e.g.*, ‘In this paper, we are going to show that …’.

**Methods/Materials/Patients.** The pitfall is making it poorly organised and superficial. This section needs to provide precise and sufficient information to allow reproducibility. A reader must be able to repeat what has been done, be it a laboratory experiment or a trial and, so, this section must be explicit. It should include information on institutions, years, institutional review board approval and participant consent, official names and sources of reagents, etc. The statistical section is of the utmost importance and should involve experts.

**Results.** The pitfall of this section is that it is frequently too short! In a sense, this is the very essence of why a paper is written: to disclose the results of the work. As such, it should be informative, consistent, and include the description of the findings in a meaningful manner. However, it should avoid being repetitive, particularly when the data are shown in detail on tables and/or figures. Thus, free text should be used to state the general result (of an experiment, a clinical comparison, etc.), referring the reader to the tables/figures for the precise details.

**Discussion.** This section’s most frequent pitfall is being the mere repetition of the Results! Instead, the final section should be the ‘brain’ of the paper! It should be the creative section about *interpretation* of the results. The discussion should begin with the most important and novel finding(s). It should include the literature review and explain how the current data differ from or are in line with previously published work. At the end, a short paragraph with the conclusions, i.e., the main ‘take home message’ of the paper, should be inserted.

**Abbreviations.** All abbreviations should be defined at first mention of the word/expression, and thereafter used only as the abbreviation. Definition of abbreviations only ‘count’ for that section, *i.e.*, they have to be given at first mention in the Title, Abstract, Body of the paper, Legends to Figures, Tables, and Supplementary text/material. Words or expressions should only be abbreviated if they are used at least 3 times in that section.

## The peer review process

When sent out for peer-review, a manuscript is evaluated by experts to determine if it fits within the scope of the journal, and to assess if the research is of sufficient novelty and quality. A manuscript can be deemed unsuitable at a first pass, and the Chief Editor can use a *rapid* rejection decision, which allows the authors to submit to another journal as quickly as possible. In contrast, if it is considered suitable, it will be reviewed by one, two or more experts. The Editor then makes an initial decision based on the reviewers’ report and will accept it straight away (extremely rare), reject it or, the most frequent outcome, ask for revisions (minor or major). If accepted, this decision is sent to the corresponding author.

The peer review process should be approached in a positive way. It is an excellent opportunity to improve a manuscript. Thus, the authors should include a cover letter with point-by-point responses and ensure that they respond to *all* comments. If the authors do not agree with a reviewer’s comment, they should explain why. Upon resubmission, a paper may be sent out for review again, and the authors may be asked to carry out further revision. Acceptance is never guaranteed until the corresponding author receives the Editor’s letter stating that the paper has been accepted for publication.

## Concluding remarks

Scientific writing is about clarity, precision, unambiguity, style, and elegance. An article must be written in the most possibly concise way, without unnecessary repetitions. Authors should avoid wasting ‘writing space’ with lengthy descriptions of data which do not add relevant information. Paying attention to this golden rule helps to avoid criticism from reviewers specifically targeting data which do not make sense and cannot be logically discussed/explained. The time, effort, discipline and hard work put into writing a good paper is amply rewarded when that work is published and made ‘forever’ available to the scientific/medical community as a source of useful information!

### AUTHORSHIP

Writing – review & editing: Junia Melo (Equal), Mohamad Mohty (Equal). Conceptualization: Mohamad Mohty (Lead). Writing – original draft: Mohamad Mohty (Lead).

### STATEMENTS AND DECLARATIONS

The authors declare no competing financial interests in relation with this work.

### ETHICAL APPROVAL

Not applicable.

### CONSENT TO PARTICIPATE/INFORMED CONSENT

Not applicable.

### CONSENT FOR PUBLICATION

Not applicable.

